# Supplementary material for: Analysis of PPARγ Signaling Activity in Psoriasis
Source: Int J Mol Sci. 2021 Aug 10;22(16):8603. doi: 10.3390/ijms22168603 (PMC8395241; doi:10.3390/ijms22168603)
Supplement: Supplementary file 1 [file ijms-22-08603-s001.zip › Supplemental materials_Analysis of PPARg signaling activity in psoriasis/Pathway models/Models images and html files/PPARG inhibits phenotypes assotiated with psoriasis/143468.html]

vasoconstriction


# Cell Process vasoconstriction

|  |  |
| --- | --- |
| URN | urn:agi-gocellproc:0042310 |
| Total Entities | 2 |
| Connectivity | 3702 |
| Name | vasoconstriction |

---

|  |  |
| --- | --- |
| ChildConcepts | blood vessel contraction |
|  | lung vasoconstriction |

---

|  |  |
| --- | --- |
| Pathway | Apoptosis of Cochlear Hair Cells (Noise-Induced, Mouse Model) |
|  | Adrenoceptors Physiological Effects |
|  | Eicosanoids in Inflammation |
|  | Mast-Cell Activation via IgE Signaling |
|  | SNS Related Adrenal Cortex Response |
|  | Vascular Endothelial Cell Activation by NO |
|  | Vascular Endothelial Cell Activation by Growth Factors |
|  | IL1B Expression Targets -> Nociception |
|  | CHRNA7 -> NOS1 Production |
|  | Vitamin C Related Norepinephrine Synthesis |
|  | Scavenger Receptor OLR1 in Inflammation Related Endothelial Dysfunction |
|  | RAGE/AGER and S100 Proteins in Cardiovascular Injury |
|  | Dilated Cardiomyopathy |
|  | Alcoholic Dilated Cardiomyopathy (Mouse Model) |
|  | Ca2+ Signaling in Dilated Cardiomyopathy |
|  | Angiotensin-Aldosterone System Activation Scheme |
|  | Endothelin Signaling in Arterial Hypertension |
|  | Aldosterone Synthesis Acute Regulation |
|  | Aldosterone Synthesis Chronic Regulation |
|  | Endothelial Cell Dysfunction in Pulmonary Hypertension |
|  | Hemolysis Associated Pulmonary Hypertension |
|  | Smooth Muscle Cell Dysfunction in Pulmonary Hypertension |
|  | Renin-Angiotensin-Aldosterone System in Myocardial ischemia |
|  | Vascular Reactivity in Raynaud Disease |
|  | Cold Stress and Adrenoceptor alpha 2C Signaling in Raynaud Disease |
|  | NO Synthesis in Early Vasospasm |
|  | Advanced Glycation End Products Pathway in Diabetic Microangiopathy |
|  | Protein Kinase C in Diabetic Microangiopathy |
|  | Peripheral Tissue Microangiopathy |
|  | Endothelial Cell Dysfunction in Progressive Diabetic Nephropathy |
|  | Microangiopathy in Diabetic Neuropathy |
|  | Proliferative Diabetic Retinopathy |
|  | Mast-Cell Activation in Asthma |
|  | Muscular Dystrophies |
|  | Nitroxyl and CGRP in Migraine Pain Activation |
|  | Folate Cycle and Homocysteine Overproduction |
|  | Vascular Smooth Muscle Cell Vasodilation in Brain |
|  | Glutamate Overdose and Aura Effect in Familial Hemiplegic Migraine Type 3 |
|  | Glutamate Overdose and Aura Effect in Migraine Type 1 |
|  | Glutamate Overdose and Aura Effect Caused by Mutations in SLC1A3 Gene |
|  | Glutamate Overdose and Aura Effect in Migraine Type 2 |
|  | Glutamate Overdose and Aura Effect Caused by Mutations in PRRT2 Gene |
|  | Glutamate Overdose and Aura Effect Overiew |
|  | NO Effect on Vasodilation and Vasoconstriction |
|  | Advanced Glycation End Products in Diabetes or Age Related Erectile Dysfunction |
|  | Endothelial Cell Dysfunction in Glomerulonephritis |
|  | Endothelial Cell Dysfunction in Pyelonephritis |
|  | Valdecoxib Induced Ischemic Disease |
|  | Cyclosporine Induced Nephrotoxicity |
|  | ROS and RNS in Vasoconstriction and Vasodilation Regulation |
|  | PPARG inhibits phenotypes assotiated with psoriasis |
|  | efects selected |
|  | 2\_The role of endothelial cells in pyelonephritis |
|  | 2\_Oxidized LDL causes endothelial dysfunction in atherosclerosis |
|  | 1\_Endothelial cells stimulate vasoconstriction and thrombosis in pulmonary hypertension |
|  | 4\_Endothelial cells repress vasoconstriction in hypertension |
|  | 1\_Rise in aldosterone synthesis in adrenal zona glomerulosa cells |
|  | 1\_Microangiopathy in diabetic neuropathy |
|  | 2\_Glutamate overload caused by mutations |
|  | 1\_2\_Glutamate overload and NO-mediated vasodilation |

---

|  |  |
| --- | --- |
| MedScan ID | 4142310 |

---

|  |  |
| --- | --- |
| Alias | vessels constrictions |
|  | vasoconstrictor reflex |
|  | vascular constrictions |
|  | constriction of the vessels |
|  | blood vessel constriction |
|  | vasoconstriction |
|  | vessels constriction |
|  | constrictions of the vessels |
|  | vasoconstrictor test |
|  | blood vessel constrictions |
|  | vascular constriction |
|  | vasoconstrictor reflexes |
|  | vessel constrictions |
|  | constriction of the vessel |
|  | vasoconstrictor tests |
|  | vasoconstrictions |
|  | constrictions of the vessel |
|  | vaso-constriction |
|  | vessel constriction |

---

|  |  |
| --- | --- |
| GO ID | 0042310 |

---
